# Impact of interfractional anatomical variation and setup correction methods on interfractional dose variation in IMPT and VMAT plans for pancreatic cancer patients: A planning study

**DOI:** 10.1002/acm2.12883

**Published:** 2020-04-30

**Authors:** Ryo Ashida, Mitsuhiro Nakamura, Michio Yoshimura, Takashi Mizowaki

**Affiliations:** ^1^ Department of Radiation Oncology and Image‐applied Therapy Graduate School of Medicine Kyoto University Kyoto Japan; ^2^ Division of Medical Physics Department of Information Technology and Medical Engineering Human Health Sciences Graduate School of Medicine Kyoto University Kyoto Japan

**Keywords:** bone‐matching vs organ‐matching, IMPT, interfractional anatomical variation, VMAT, pancreatic cancer

## Abstract

**Purpose:**

To investigate the impact of interfractional anatomical changes and setup correction methods on dose distributions in pancreatic cancer patients under breath‐hold conditions.

**Methods:**

Three intensity‐modulated proton therapy (IMPT) plans with different beam arrangements and one volumetric‐modulated arc therapy (VMAT) plan prescribing 54 Gy in 30 fractions were created for 10 patients who underwent three additional CT scans performed at an interval of 1–2 weeks. The additional CT sets were rigidly registered to the simulation CT set using both bone‐matching (BM) and organ‐matching (OM) methods in each patient. Recalculated dose distributions and dose–volume indices on the additional CT sets using either the BM or the OM method were compared with the simulation values.

**Results:**

Differences in the gross tumor volume D_98%_ value from the simulation sets ranged from −0.8 to −5.9% on average. In addition, the variations were larger with OM compared with BM for two IMPT plans. Meanwhile, differences in the D_98%_ value in the region isotropically enlarged by 5 mm from the gross tumor volume were significantly improved with OM on two IMPT plans and the VMAT plan. Among the organs at risk, the dose–volume indices were significantly improved with OM only in the duodenum on all plans.

**Conclusion:**

Organ‐matching may be a better setup correction technique than BM for both photon therapy and IMPT plans. However, in some beam arrangements of IMPT, the dose distribution may be somewhat worse using OM, due to interfractional anatomical variation. Therefore, it is important to choose beam angles that are less likely to be influenced by changes in the gastrointestinal gas volume, especially in IMPT plans.

## 1 INTRODUCTION

Pancreatic cancer is one of the most common causes of cancer‐related mortality.^1^ Although the most promising treatment method for long‐term survival of pancreatic cancer patients is surgical resection, at diagnosis most patients present with inoperable disease, such as locally advanced or metastatic disease; therefore, radiotherapy is considered an important treatment modality, particularly for patients with locally advanced pancreatic cancer (LAPC).^2^

There are some difficulties associated with treating LAPC patients with radiotherapy. Anatomical positioning causes a significant challenge, as the pancreas is surrounded by radiosensitive organs at risk (OARs) such as the stomach and duodenum. This makes it difficult to deliver high doses to the tumor without increasing the radiation dose to OARs. Intensity‐modulated radiotherapy (IMRT), volumetric modulated arc therapy (VMAT), and particle therapy have been effective in reducing the dose to surrounding OARs in simulation.^3^, ^4^, ^5^ However, as several investigators have mentioned, there are issues with position repeatability when treating the pancreas, due to intrafractional body movements, respiratory motion, and variations in gastrointestinal contents.^6^, ^7^ The positional uncertainty results in a decreased target coverage and increased dose to OARs. To reduce these uncertainties, immobilization devices, respiratory motion management, and fasting before radiotherapy have been commonly employed.^8^, ^9^

Among the setup correction methods used before beam delivery are bone‐matching (BM) and organ‐matching (OM) methods. With respect to the setup correction method, it has been suggested that marker‐matching and OM are far superior to skin‐marking‐matching methods and superior even to BM in photon therapy.^10^ Likewise, in carbon ion therapy (CIRT), OM and marker‐matching are reportedly superior to BM for the lung and liver.^11^, ^12^ Meanwhile, in CIRT for pancreatic cancer, a small effect of the setup correction method on dose distribution was demonstrated^13^; this is because the dose distribution in particle therapy, compared with photon therapy, is easily distorted by interfractional anatomical variations such as changes in physique and gastrointestinal gas in abdominal therapy.^14^

The interfractional dose variations due to differences in setup correction methods in CIRT were assessed in previous studies.^11^, ^12^, ^13^ However, there is insufficient literature on quantitative evaluation of the dose variations among setup correction methods in proton beam therapy (PBT). Furthermore, the interfractional dose variation of different radiotherapy modalities using different setup correction methods has not been investigated previously in pancreatic cancer patients.

The purpose of this study was to investigate the impact of interfractional anatomical variation and setup correction methods, and specifically OM (i.e., cone‐beam CT [CBCT] matching) and BM (i.e., orthogonal kV x‐ray imaging matching), on the dose distributions in pancreatic cancer patients under end‐exhalation breath‐hold (EBH) conditions.

## 2 MATERIALS AND METHODS

This study was conducted in accordance with the Declaration of Helsinki and was approved by our Institutional Ethical Review Board (approval number R1446).

### 2.A Patients and CT scans

Data from 10 consecutive LAPC patients treated with chemoradiotherapy at our institution between January 2009 and August 2009 were used in this study. All patients had undergone three additional CT scans, performed at an interval of 1–2 weeks during a chemoradiotherapy course and under the same conditions as in the simulation CT scan. The characteristics of the patients are shown in Table 1.

**Table 1:** Patient characteristics.

Pt. no.	Sex	Age (yr)	Location	TNM
1	F	76	Body	T4N0M0
2	F	72	Body	T4N0M0
3	M	44	Body	T4N1M0
4	M	72	Body	T4N0M0
5	M	66	Head	T4N0M0
6	M	58	Head	T3N0M1 (LYM*)
7	M	66	Body	T4N0M0
8	M	66	Head	T4N0M0
9	M	66	Head	T4N0M0
10	M	46	Body	T4N0M0

Note

Pt. no., patient number; M, male; F, female; location, location of the tumor in the pancreas; TNM, stage according to the International Union Against Cancer classification 7th edition; *, distant lymph node within the irradiation field.

CT was performed under EBH condition using the LightSpeed RT scanner (GE Healthcare, Little Chalfont, UK) and Real‐time Position Management system (RPM; Varian Medical Systems, Palo Alto, CA). The CT slice thickness was 2.5 mm. Patients fasted for at least 3 h and were immobilized in the supine position with both arms raised in a BodyFIX vacuum cushion (Elekta, Stockholm, Sweden).

### 2.B Target volume and OAR delineation

The gross tumor volume (GTV) included the primary tumor and metastatic lymph nodes. The clinical target volume (CTV) was defined as the GTV plus a 5 mm margin in each direction (GTV+5 mm) combined with the retropancreatic and para‐aortic space between the 10 mm superior of the celiac axis and the 10 mm inferior of the superior mesenteric artery. The planning target volume (PTV) was defined by adding a 5 mm margin to the CTV.^5^, ^15^, ^16^, ^17^ The same PTV was used in the intensity‐modulated proton therapy (IMPT) and volumetric‐modulated arc therapy (VMAT) plans, based on previous studies in which 5 mm PTV margins were used even in IMPT and CIRT plans.^5^, ^16^, ^17^ In addition, using the same structures yielded equivalent plans for IMPT and VMAT. The GTV, GTV+5 mm, stomach, duodenum, and small intestine were delineated on all 40 CT sets (10 simulation and 30 additional CT sets) and CTV, PTV, spinal cord, liver, and kidney were delineated on 10 simulation CT sets by one radiation oncologist and reviewed by two experts.

### 2.C Treatment planning

**Fig. 1:**
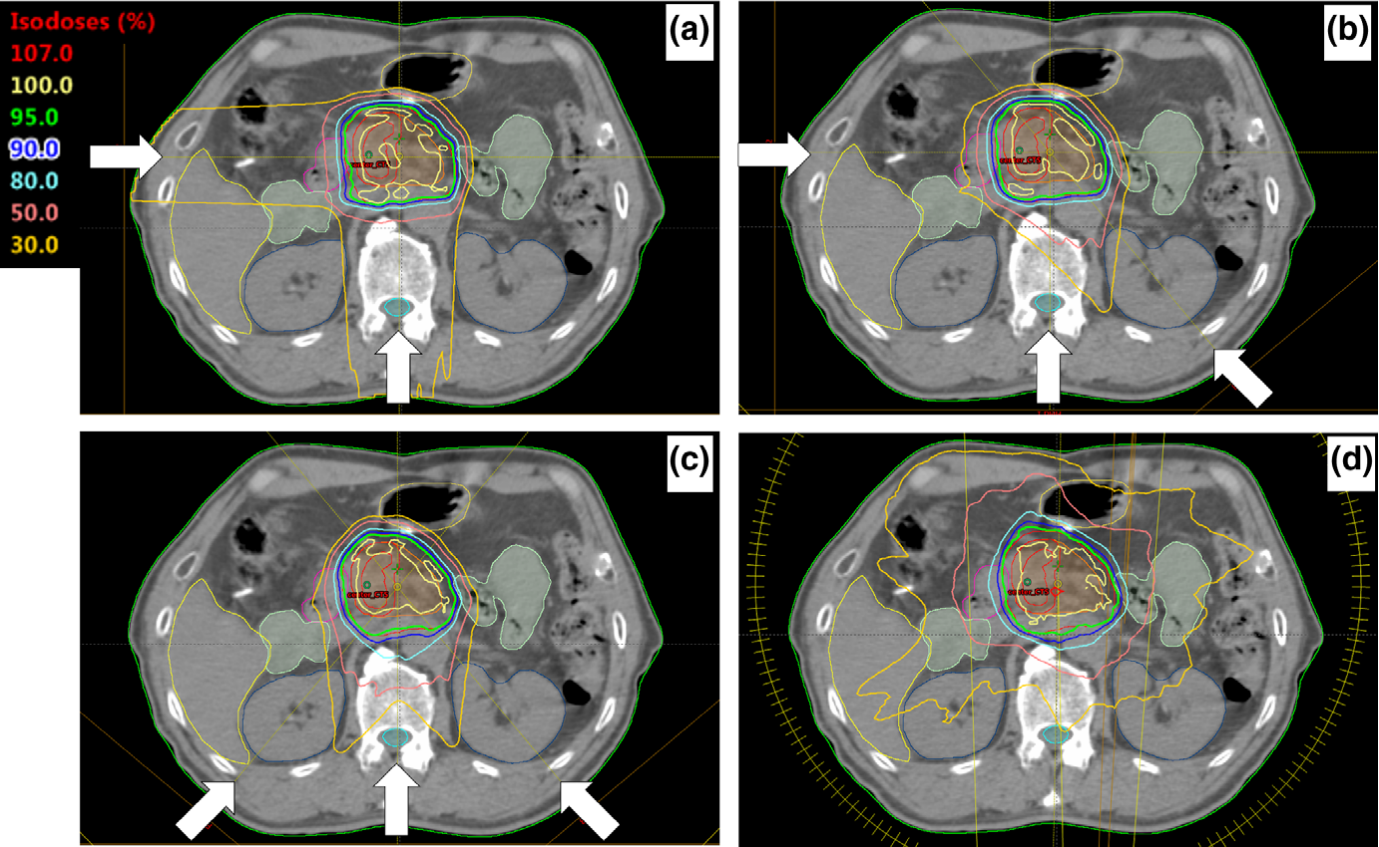
Field setup and dose deposition. (a) Proton plan with right and posterior beams (RP plan), (b) proton plan with right, posterior and left posterior oblique beams (RPOL plan), (c) proton plan with right posterior oblique, posterior and left posterior oblique beams (ORPOL plan), (d) photon plan (volumetric‐modulated arc therapy plan).

We constructed three different IMPT plans and one VMAT plan prescribing 54 Gy in 30 fractions for each patient on the Eclipse treatment planning system, version 15.6 (Varian Medical Systems) for the purpose of this study. In the IMPT plans, we used a spot‐scanning technique with two or three incident proton beams of kinetic energies between 70 and 250 MeV delivered by the Varian proton therapy system. Three different IMPT plans were used: (a) gantry angles of 180° and 270° (right and posterior [RP] plan); (b) gantry angles of 135°, 180°, and 270° (right, posterior, and left posterior oblique [RPOL] plan); (c) gantry angles of 135°, 180°, and 225° (right posterior oblique, posterior, and left posterior oblique [ORPOL] plan). Since a previous study showed that the dose distributions from the anterior and left directions may be overshot/undershot,^18^ the combination of right and posterior directions has since become a commonly used beam angle in two‐field IMPT planning.^19^ The RPOL plan was designed to add a third beam path other than anterior and left directions to the RP plan. The beam angles of the ORPOL plan are also commonly used in IMPT plans in three‐field IMPT planning.^19^, ^20^ The advantage is a reduction of the volume delivered to the descending part of the duodenum via the beam path. The VMAT plan consisted of one coplanar full‐arc of 10 MV flattening filter‐free photon beams that rotated clockwise from 181° to 179° using the TrueBeam STx with a high‐definition 120‐leaf multileaf collimator (Varian Medical Systems). The dose calculation algorithms used were the analytical proton convolution superposition algorithm for the IMPT plans and Acuros XB (Varian Medical Systems) for the VMAT plan. The CT numbers of gastrointestinal gas were not overridden to those of the surrounding tissue in this study. Figure 1 illustrates the field setup and dose distribution.

The prescribed dose of 54 Gy was the dose to 50% of the PTV (D_50%_: D_X%_ is the dose delivered to X% of the target volume). The maximum dose should be <60 Gy. Regarding the OARs, the V_45Gy_ (V_XGy_ is the volume receiving X Gy), V_48Gy_, and V_52Gy_ of the stomach, duodenum, and small intestine were limited to <20, 5, and 1 cm^3^, respectively. Although a PTV D_95%_ ≥ 95% was the goal (no deviation), if this was difficult to achieve within the constraints of the gastrointestinal tract, the PTV was reduced to D_95%_ ≥ 90% (minor deviation). The constraints of OARs other than the gastrointestinal tract were as follows: maximum dose to the spinal cord <45 Gy and to each kidney V_18Gy_ < 30%, and mean dose to the liver < 25 Gy.

### 2.D Analysis of interfractional dose variation

The additional CT sets were rigidly registered to the simulation CT sets using both BM and OM, and the target volumes and OARs were again delineated on the additional CT sets. OM was conducted by a single board‐certified radiation oncologist using three‐dimensional translation without rotation. Then, the plans on the simulation CT sets were assigned to the additional CT sets and recalculated in the BM and OM settings without overriding the CT numbers of gastrointestinal gas to evaluate the interfractional dose variations, respectively (assigned plan). To evaluate the dose coverage, the D_50%_ and D_98%_ of the GTV and GTV+5 mm in the assigned plans were evaluated. For the OARs, the V_45Gy_, V_48Gy_, and V_52Gy_ of the stomach, duodenum, and small intestine were evaluated, respectively.

All statistical analyses were performed using R (version 3.4.3; the R Foundation for Statistical Computing, Vienna, Austria). The dose–volume indices (DVIs) were compared between BM and OM using the paired t‐test, and a *P* < 0.05 was considered to indicate statistical significance.

## 3 RESULTS

**Fig. 2:**
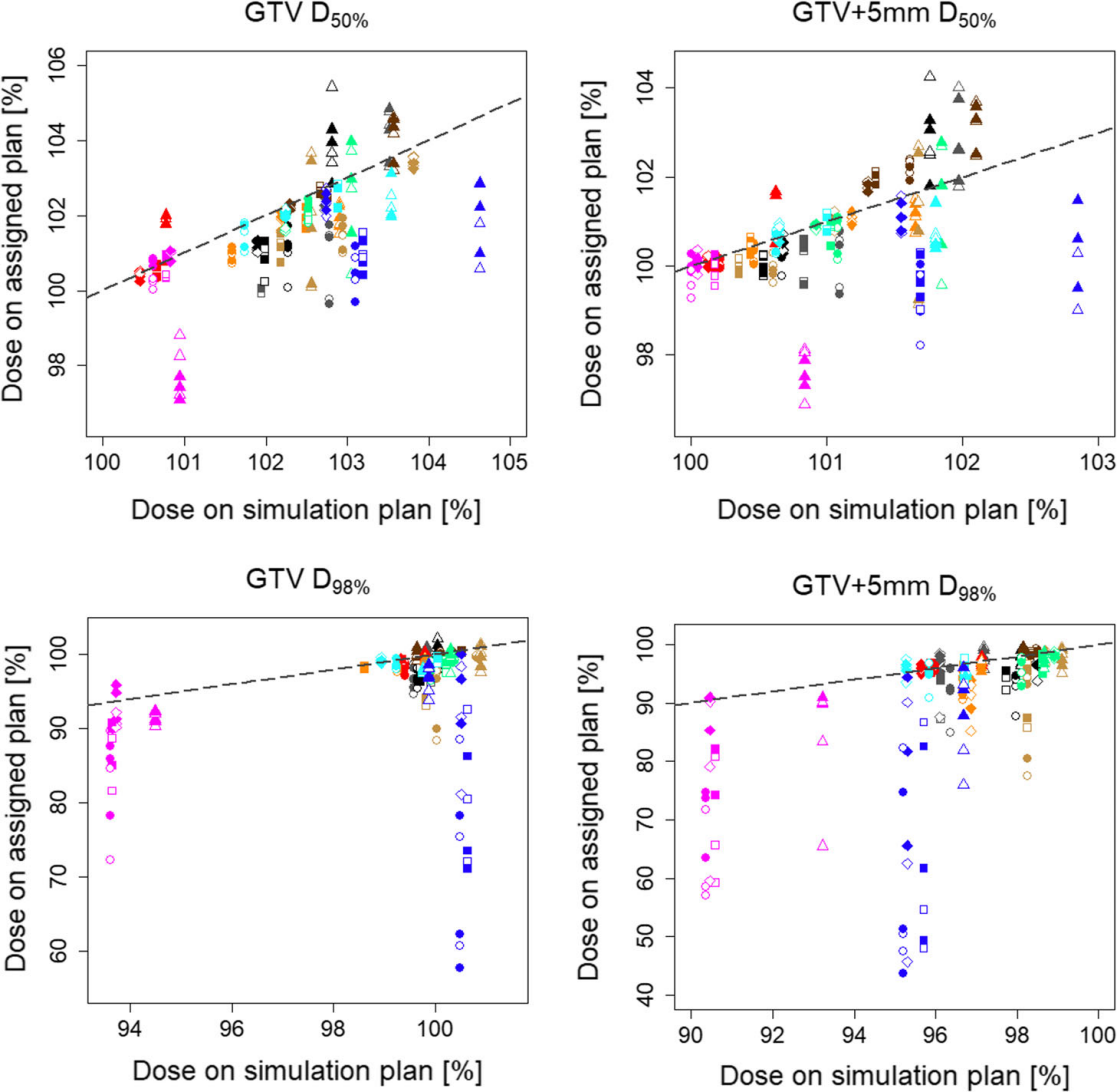
Differences in the dose–volume indices (DVIs) of the target volumes between the simulation plans and the assigned plans. The horizontal and vertical axes show the DVIs for the simulation plans and assigned plans, respectively. The four symbol shapes show the plan type (circle: RP plan, square: RPOL plan, rhombus: ORPOL plan, triangle: volumetric‐modulated arc therapy plan), and the 10 colors indicate the 10 patients (empty: with bone‐matching, filled: with organ‐matching). The chain line is the line at which the DVI on the simulation plans versus the assigned plans is the same.

**Fig. 3:**
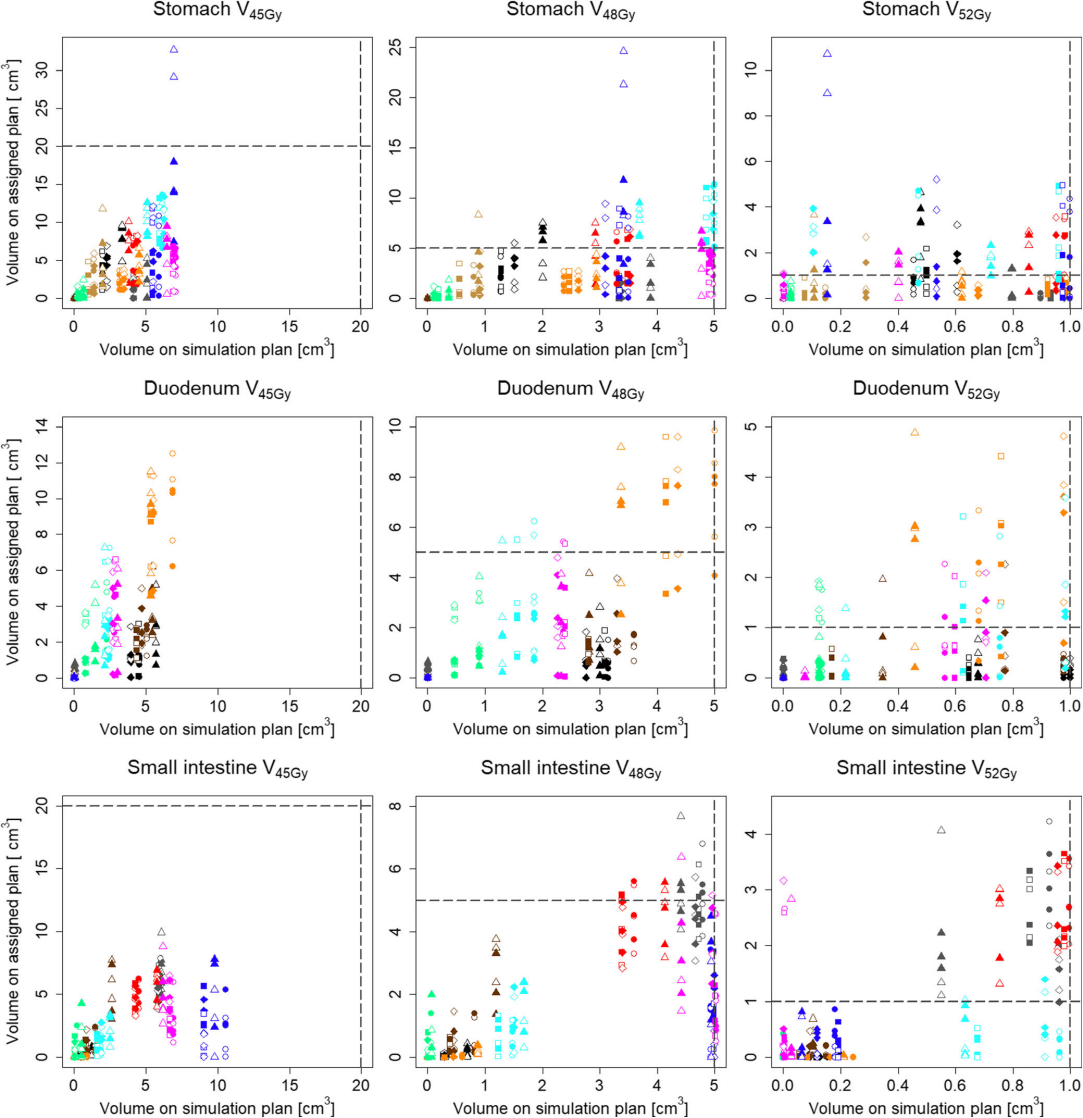
Differences in the dose–volume indices (DVIs) of organs at risk between the simulation plans and the assigned plans. The horizontal and vertical axes show the dose–volume indices for the simulation plans and assigned plans, respectively. The four symbol shapes indicate the plan type (circle: RP plan, square: RPOL plan, rhombus: ORPOL plan, triangle: volumetric‐modulated arc therapy plan), and the 10 colors the 10 patients (empty: with bone‐matching, filled: with organ‐matching). The chain line shows the dose–volume constraints in the simulation.

In the simulation plans, 2 of the 10 patients required minor deviations in the PTV D_95%_ due to OARs close to the PTV and  difficulty to comply the dose constraints; however, the other dose–volume constraints were all met. The DVIs of the simulation plans and assigned plans are shown in Figs. 2 and 3.

**Fig. 4:**
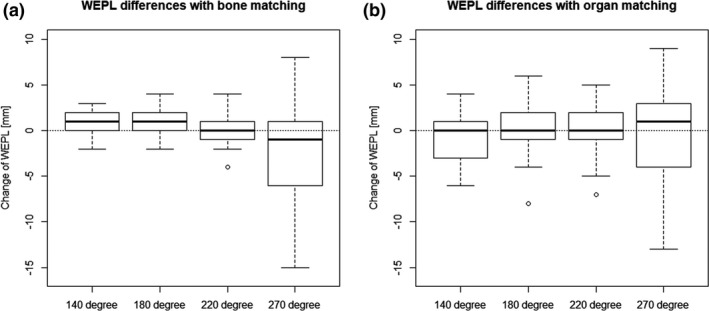
Differences in the water equivalent path length (WEPL) with bone matching (BM) and organ matching (OM). The boxplots show the differences from simulation plans in the WEPL at isocenter among each gantry angle (a) with BM, and (b) with OM.

The positional differences (mean ± standard deviation [SD]) between BM and OM were 1.4 ± 2.3 (range, −3.1 to 5.8), 0.4 ± 1.7 (range, −3.2 to 4.4) and −0.2 ± 3.6 (range, −7.2 to 10.6) mm in the lateral, vertical, and longitudinal directions, respectively. Positive values indicate that OM showed more couch movement to the left, posterior, and superior directions than did BM. Differences of the water equivalent path length (WEPL) at isocenter from simulation plans (mean ± SD) in 140°, 180°, 220° and 270° fields were 0.5 ± 1.4 (range, −2.0 to 3.0), 0.9 ± 1.8 (range, −2.0 to 4.0), 0.2 ± 1.6 (range, −4.0 to 4.0) and −2.3 ± 5.1 (range, −15.0 to 8.0) mm with BM, and −0.7 ± 2.5 (range, −6.0 to 4.0), 0.2 ± 2.8 (range, −8.0 to 6.0), 0.2 ± 2.7 (range, −7.0 to 5.0) and −0.4 ± 5.2 (range, −13.0 to 9.0) mm, respectively (Fig. 4). Positive values indicate that the WEPL increased in assigned plans. These values show that the 270° field had more deviation than other fields; particularly, student’s t‐test showed that there were significant differences with BM (*P* < 0.05).

**Fig. 5:**
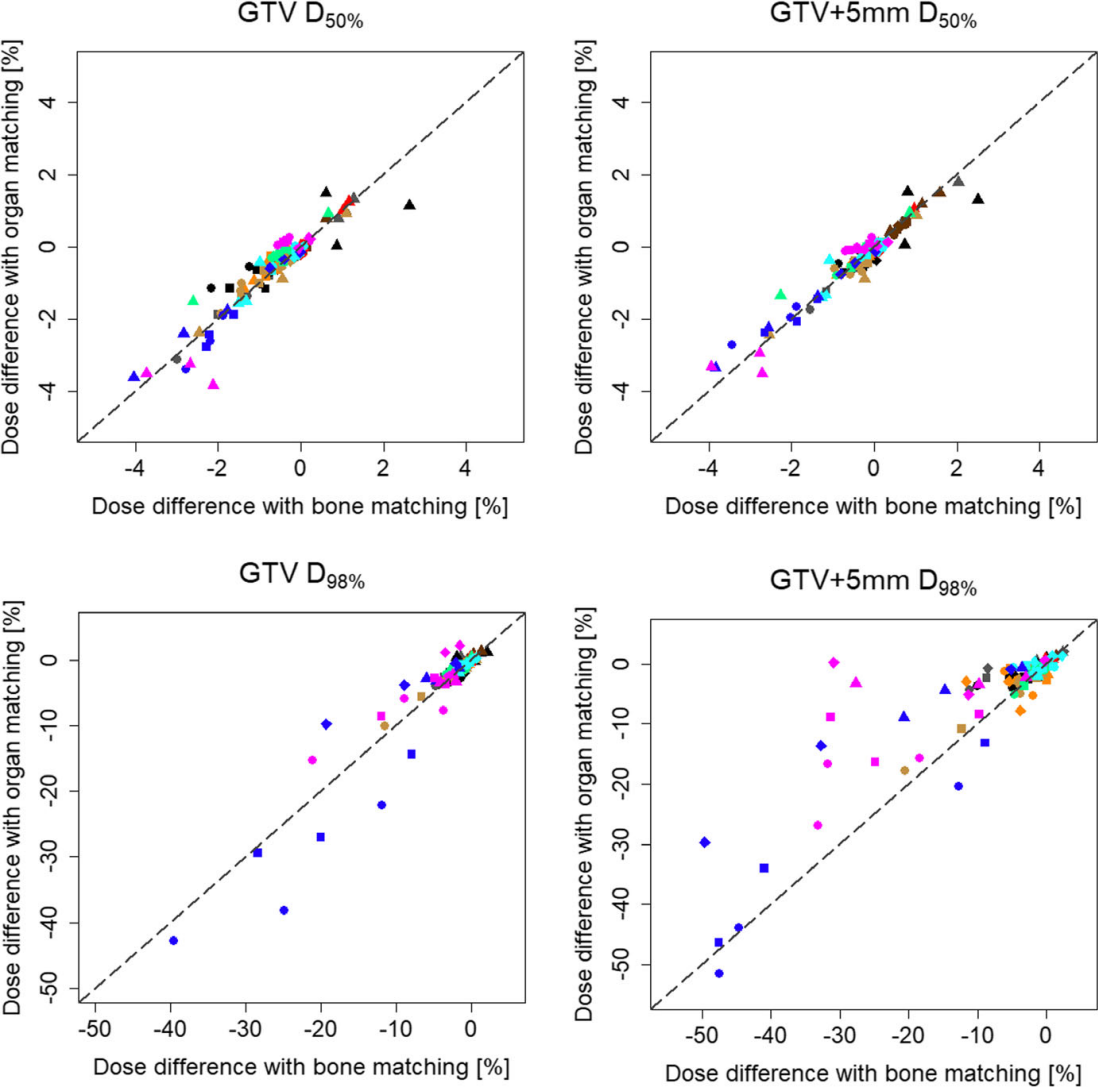
Differences in the DVIs of the target volumes between bone matching (BM) and organ matching (OM). The horizontal and vertical axes show the differences obtained using BM and OM, respectively. The four symbol shapes indicate the plan type (circle: RP plan, square: RPOL plan, rhombus: ORPOL plan, triangle: VMAT plan), and the 10 colors the 10 patients. The chain line is the line at which the dose difference using BM versus OM is the same.

The differences (mean ± 2 SD) in the DVIs between the simulation and assigned plans are shown in Table 2. Figure 5 shows the differences in the D_50%_ and D_98%_ of the GTV and GTV + 5 mm using OM vs BM. The differences in both GTV and GTV + 5 mm D_50%_ were within ±1% on average in all IMPT and VMAT plans for both setup correction methods. However, those of D_98%_ varied between the plans, ranging from −0.8% to −5.9% on average. The tendency of a larger deviation from zero of the mean differences in the GTV and GTV+5 mm D_98%_ was greater in the RP and RPOL plans than in the ORPOL and VMAT plans. In addition, the two SD values of the differences were >10% in the RP and RPOL plans, even for GTV D_98%_. Comparing BM and OM, the difference in the GTV D_98%_ was significantly improved with OM in the VMAT plan, and that of the GTV+5 mm D_98%_ was improved in the RPOL, ORPOL, and VMAT plans (*P* < 0.05).

**Table 2:** Differences in dose–volume indices (mean ± 2 SD) between the simulation and assigned plans.

	RP		RPOL		ORPOL		VMAT	
	BM − Sim	OM − Sim	BM − Sim	OM − Sim	BM − Sim	OM − Sim	BM − Sim	OM − Sim
GTV								
D_50%_ [%]	‐0.9 ± 1.7	‐0.8 ± 1.9*	‐0.7 ± 1.4	‐0.7 ± 1.5	‐0.3 ± 0.6	‐0.2 ± 0.5	‐0.6 ± 3.3	‐0.6 ± 3.2
D_98%_ [%]	‐5.2 ± 17.8	‐5.9 ± 21.1	‐3.7 ± 12.6	‐3.9 ± 14.5	‐1.7 ± 7.5	‐1.0 ± 4.0	‐1.2 ± 3.6	‐0.8 ± 2.7*
GTV + 5 mm								
D_50%_ [%]	‐0.5 ± 1.7	‐0.4 ± 1.5	‐0.3 ± 1.4	‐0.3 ± 1.4	0.0 ± 0.6	0.0 ± 0.5*	‐0.5 ± 3.4	‐0.5 ± 3.1
D_98%_ [%]	‐9.2 ± 26.8	‐7.9 ± 25.9	‐7.1 ± 25.0	‐5.4 ± 21.0*	‐5.5 ± 23.6	‐2.1 ± 12.1*	‐3.1 ± 13.3	‐1.0 ± 4.2*
Stomach								
V_45Gy_ [cm^3^]	0.0 ± 5.9	‐0.4 ± 4.8	0.2 ± 6.2	‐0.1 ± 4.7	0.4 ± 6.3	0.1 ± 5.0	2.9 ± 13.6	1.8 ± 7.0
V_48Gy_ [cm^3^]	0.0 ± 4.8	‐0.4 ± 3.8	0.2 ± 5.1	‐0.2 ± 3.7	0.4 ± 5.4	0.1 ± 4.1	2.3 ± 11.0	1.3 ± 5.5
V_52Gy_ [cm^3^]	0.4 ± 2.5	0.1 ± 1.9	0.5 ± 2.6	0.1 ± 1.9	0.7 ± 3.0	0.4 ± 2.1	1.3 ± 5.1	0.7 ± 2.3
Duodenum								
V_45Gy_ [cm^3^]	0.2 ± 4.7	‐0.5 ± 3.7**	0.3 ± 4.5	‐0.3 ± 3.6**	0.4 ± 4.1	‐0.3 ± 3.4**	0.5 ± 4.6	‐0.3 ± 3.9**
V_48Gy_ [cm^3^]	0.2 ± 3.9	‐0.3 ± 2.9**	0.3 ± 3.7	‐0.2 ± 2.9**	0.4 ± 3.5	‐0.2 ± 2.8**	0.5 ± 3.7	‐0.2 ± 2.9**
V_52Gy_ [cm^3^]	0.2 ± 1.8	‐0.1 ± 1.1**	0.5 ± 2.0	0.1 ± 1.2**	0.4 ± 2.2	0.1 ± 1.6**	0.4 ± 2.0	0.1 ± 1.4**
Small intestine								
V_45Gy_ [cm^3^]	‐1.4 ± 6.1	‐1.2 ± 5.1	‐1.1 ± 5.1	‐0.9 ± 4.0	‐1.1 ± 5.1	‐0.8 ± 3.7	‐0.5 ± 5.9	‐0.2 ± 4.1
V_48Gy_ [cm^3^]	‐0.8 ± 3.4	‐0.7 ± 3.0	‐0.7 ± 3.3	‐0.6 ± 2.8	‐0.6 ± 3.1	‐0.5 ± 2.3	‐0.2 ± 3.5	‐0.1 ± 2.4
V_52Gy_ [cm^3^]	0.4 ± 2.2	0.4 ± 1.9	0.4 ± 1.8	0.3 ± 1.6	0.3 ± 1.6	0.2 ± 1.2	0.4 ± 2.0	0.3 ± 1.4

*Abbreviations*: BM – Sim, difference between the simulation plans and plans assigned using bone‐matching; GTV, the gross tumor volume, GTV + 5 mm, the volume after adding a 5 mm margin in all directions to the GTV, DX%, the dose to X% of the target volume; OM – Sim, difference between the simulation plans and plans assigned using organ‐matching; ORPOL, proton plans with right posterior oblique, posterior and left posterior oblique beams, VMAT, volumetric‐modulated arc therapy; RP, proton plans with right and posterior beams; RPOL, proton plans with right, posterior and left posterior oblique beams; SD, standard deviation; V_XGy_, the volume receiving X Gy.

* p < 0.05, paired *t*‐test.

** p < 0.01, paired *t*‐test.

**Fig. 6:**
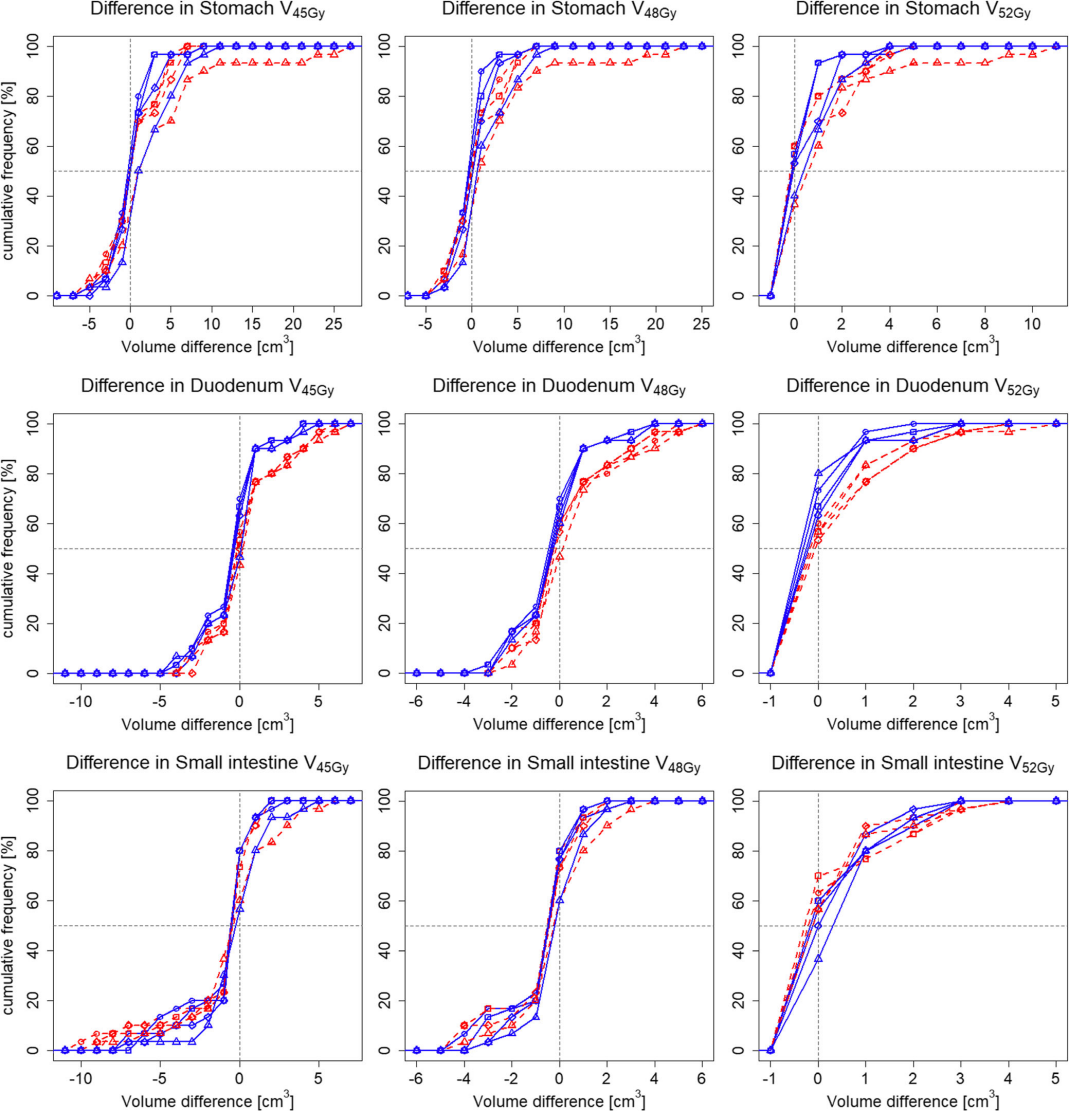
Cumulative frequency of dose–volume differences in organs at risk between assigned and simulation plans. The four symbol shapes show the plan type (circle: RP plan, square: RPOL plan, rhombus: ORPOL plan, triangle: VMAT plan). The red dashed versus blue solid lines show the data obtained using bone matching versus organ matching, respectively. The dotted line shows no change in dose–volume indices in the horizontal axis and 50% change in the vertical axis.

Regarding the OARs, the VMAT plan caused larger interfractional variations in the stomach V_45Gy_ and V_48Gy_ than did the three IMPT plans. No notable differences in the DVIs in the duodenum or small intestine were observed among the plans. Although the average difference in the DVI in the duodenum was within ±1 cm^3^ for BM and OM, the DVIs were significantly improved by OM on all plans (*P* < 0.01). Graphs of the cumulative frequencies showing the differences in the V_45Gy_, V_48Gy,_ and V_52Gy_ of the stomach, duodenum, and small intestine are shown in Fig. 6. While the graphs should ideally be in the shape of a step (as planned) or show a shift to the negative side (better in the assigned plans), the graphs tended to deviate more with BM than with OM.

## 4 DISCUSSION

To the best of our knowledge, this is the first report to investigate the impact of setup correction methods on interfractional dose variation among multiple IMPT and VMAT plans in pancreatic cancer patients under EBH conditions. We found that DVIs of assigned plans for pancreatic cancer tended to improve more with OM than with BM, except in some cases. Compared with the simulation plan, the GTV+5 mm D_98%_ was reduced in the assigned plans, but the magnitude was significantly smaller with OM than with BM on the RPOL, ORPOL and VMAT plans. This indicates that OM may be useful not only for photon therapy but also for PBT with selected beam paths. The duodenum also showed significant DVI differences between BM and OM on all plans, in contrast to only small DVI differences between the IMPT and VMAT plans. The duodenum is a radiosensitive organ, and toxicity in the duodenum has been reported during treatment of pancreatic cancer; this significant dose reduction with OM is useful to reduce toxicity. Meanwhile, the stomach and small intestine showed no significant differences between BM and OM. Bowel gas and gastrointestinal contents vary with time,^9^, ^18^ and changes in the stomach and small intestine shapes due to these variations lead to both increases and decreases in the DVIs for each CT set.

**Fig. 7:**
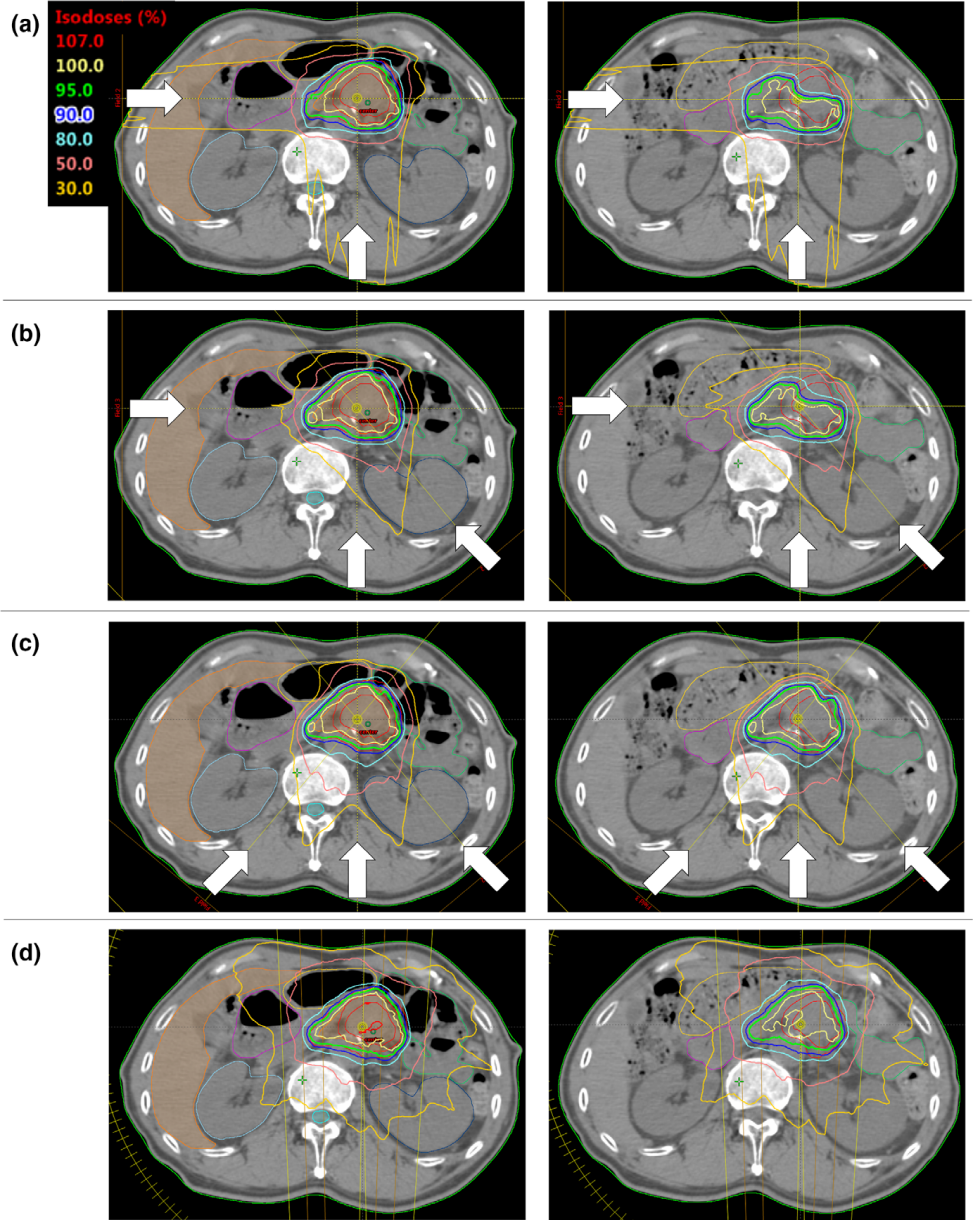
Change in dose distribution. Comparison of the dose distribution between the original plan (left) and the plan assigned to the first additional CT (right) in (a) RP, (b) RPOL, (c) ORPOL and (d) VMAT plans.

**Fig. 8:**
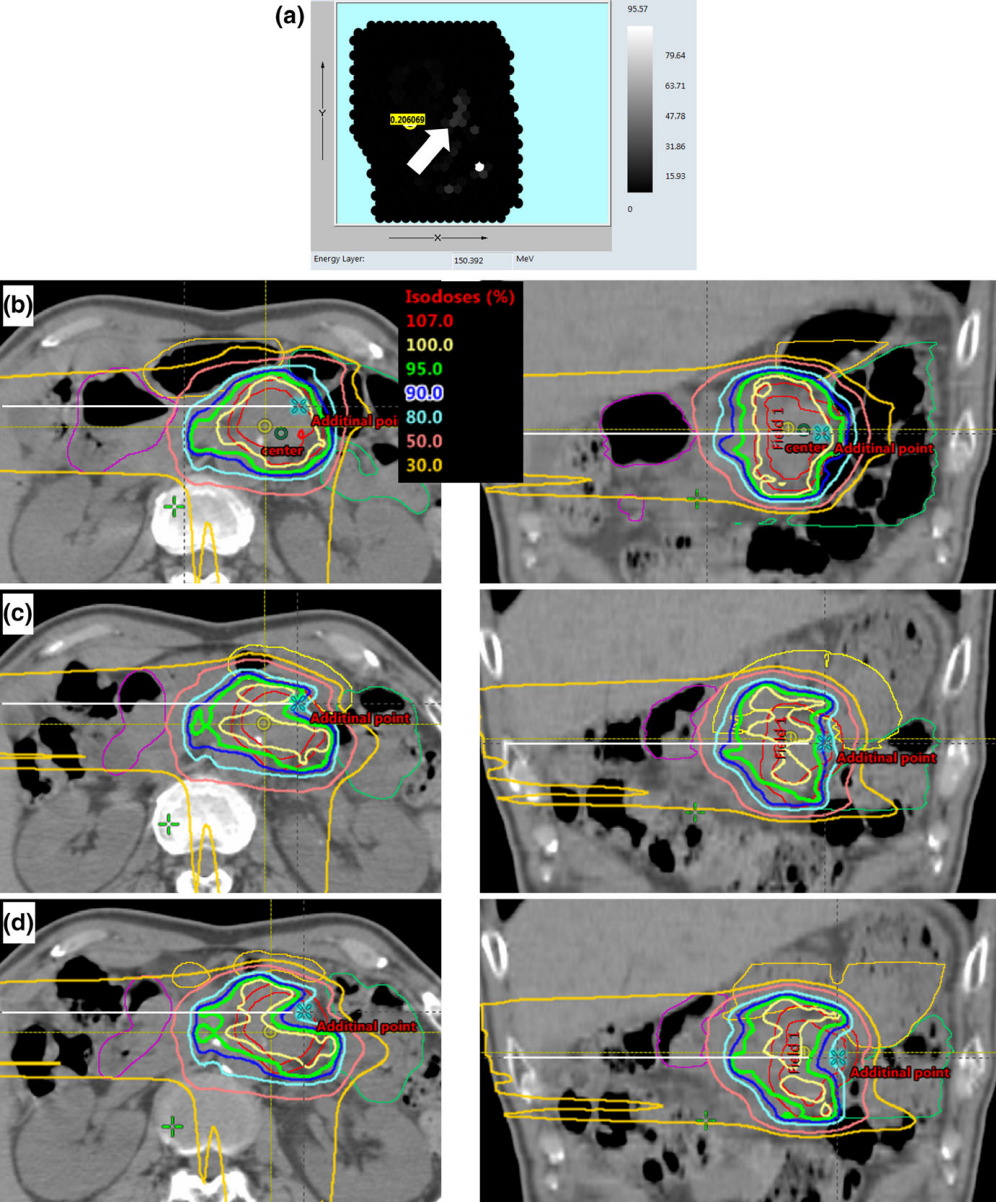
A notable example of the effect of beamlet intensity on the RP plan. The RP plan had a worse dose distribution with OM than with BM. (a): The target beamlet position on the intensity weight map (arrowed position). (b)–(d): Axial and coronal planes of the target beamlet path on the simulation plan, the assigned plan with BM and that with OM, respectively. The white lines indicate the target beamlet path line and the crosses show the additional points the water equivalent path length measured in this notable patient, 17 mm left, 10 mm anterior, and 2.5 mm inferior from isocenter.

As expected, positional differences were different between BM and OM, especially in the longitudinal direction. The SD values indicated that extreme deviations sometimes occurred, and 3 of 30 additional CT sets showed differences of >5 mm. These deviations were considered to derive from EBH failure.^6^ Pancreatic cancer showed systematic deviation in the lateral direction. From this finding, the target dose was considered better with OM compared with BM; however, this did not apply to all plans. Positional differences between the tumor and bony anatomy may result in both source‐to‐surface distance variations and variations in anatomical structures on beam paths. Proton dose distributions are not appreciably affected by source‐to‐surface distance but are affected by perpendicular shifts in the beam direction and by anatomical variations.^21^ Organ‐matching could reduce the perpendicular shifts of targets and OARs owing to more precise position repeatability against breath‐hold position deviations. And for anatomical variations, a high robustness of VMAT for interfractional anatomical changes and considerable deviations between the planned and accumulated doses in PBT has been reported in pancreatic cancer patients.^14^ This supported our finding that the interfractional dose differences in the GTV+5 mm D_98%_ were approximately 50% or less in the VMAT plan, compared with all proton plans using BM and OM (Table 2). As notable examples of dose distribution distortion, a decrease of more than 20% in the GTV+5 mm D_98%_ was observed in all plans using BM for some patients. The representative dose distributions for one of these patients are shown in Fig. 7. In this case, the dose–volume constraints were all met in each plan; however, GTV+5 mm D_98%_ greatly decreased in all assigned plans with BM and in assigned IMPT plans with OM. When comparing the simulation CT set and additional CT sets of this patient, in addition to tumor movement to the left side, the gastrointestinal gas on the simulation CT decreased somewhat on the additional CTs. It has been reported that one of the largest interfractional anatomical deviations in pancreatic cancer patients is the change in the gastrointestinal gas volume.^13^ The effect of changes in the gastrointestinal gas volume on dose distribution has been reported for CIRT over the duration of one fraction, and a reduction in target coverage was demonstrated. Kumagai et al. have reported that the distortion of the dose distribution due to gastrointestinal gas volume variations appeared mainly on the beam from the anterior and left side of the patients;^18^ however, distortion of the dose distribution was also observed on the beam from the right side in our study. Abdominal organs other than the gastrointestinal tract showed little deviation in electron density; however, gastrointestinal gas can reduce the WEPL greatly. In the cases with worse DVIs in the RP and RPOL plans using OM than BM, gastrointestinal gas surrounded the tumor on the simulation CT but little gas was present on the assigned CT set (Fig. 8). In this patient, the WEPL difference from simulation plan at additional point on high‐intensity beamlet of 270° field was quite deviated than mean difference of 10 patients at isocenter and larger with OM (30 mm) than BM (21 mm). A large WEPL change with both BM and OM could be avoided using the ORPOL plan owing to less gastrointestinal gas on beam paths. Thus, the gastrointestinal gas has a considerable effect on the dose distribution, and it is difficult to predict the location of gastrointestinal gas during the course of treatment. If in the simulation plan the CT numbers of the gastrointestinal gas were overridden to match those of the surrounding tissue, a shorter WEPL due to gastrointestinal gas could lead to an overdose in OARs at actual irradiation.

Changes in body habitus over the treatment course would also result in a change in the WEPL; therefore, replanning in IMPT patients will allow adjustments due to these changes. However, changes in WEPL due to gastrointestinal gas are difficult to evaluate, particularly using the BM method, including the use of orthogonal kV x‐ray images. Thus, an OM such as CBCT is considered preferable to evaluate WEPL during actual irradiation.

In this study, EBH was used for respiratory motion management because it provides a high interfractional reproducibility of the pancreatic tumor position.^6^ While in all IMPT and VMAT plans under EBH irradiation more than one breath‐hold will be necessary, a high intrafractional reproducibility of the pancreatic tumor position under multiple EBH using RPM has been reported.^8^ Therefore, the intrafractional dose variation derived from multiple EBH was considered to be smaller than that resulting from setup correction methods.

There were several limitations to this study. First, only ten patients were evaluated. However, even with this small number of cases, significant differences in DVIs between the simulation and assigned IMPT plans could be determined. Additionally, these differences were dependent on the setup correction method. Second, we did not use deformable image registration (DIR) to evaluate dose accumulation but evaluated the dose distribution at certain time points. However, registration uncertainties in DIR result in inaccurate deformed delineations and dose distributions,^22^ and there are advantages to using additional CT instead of daily CBCT to reduce delineation uncertainties, due to the poor soft‐tissue contrast on CBCT images. Third, we did not use robust optimization in the IMPT plans, which is suggested to protect against positional errors and range uncertainty by optimizing the sharp dose fall off to OARs.^21^ However, we did address positional error via setup correction methods. As mentioned above, the dose distribution tended to be better with OM compared with BM; therefore, the positional error was addressed effectively. However, in some cases, residual distortion of the dose distribution with OM was noted, particularly on the RP and RPOL plans. Moreover, the dose distributions using OM were even worse. The proposed robustness of IMPT is not achievable with positional repeatability alone. In the case of range uncertainty, the WEPL changes readily and significantly with interfractional anatomical variations such as gastrointestinal gas as shown in Fig. 6, and it is doubtful whether the robust optimization results were superior with VMAT plans compared with IMPT plans.

## 5 CONCLUSION

Organ‐matching may be a better setup correction technique than BM for both photon therapy and IMPT plans. However, in some beam arrangements of IMPT, the dose distribution may be somewhat worse using OM due to interfractional anatomical variation. Therefore, it is important to choose beam angles that are less likely to be influenced by changes in gastrointestinal gas volume, especially when IMPT plans are used.

## CONFLICT OF INTEREST

The authors have no conflict of interest to declare.
